# Enhanced Oil Recovery in a Co-Culture System of *Pseudomonas aeruginosa* and *Bacillus subtilis*

**DOI:** 10.3390/microorganisms12112343

**Published:** 2024-11-16

**Authors:** Dingyu Kang, Hai Lin, Qiang Li, Nan Su, Changkun Cheng, Yijing Luo, Zhongzhi Zhang, Zhiyong Zhang

**Affiliations:** 1 State Key Laboratory of Heavy Oil Processing, China University of Petroleum, Beijing 102249, China; kang_dingyu@163.com (D.K.); linhaiqh@petrochina.com.cn (H.L.); carole66@163.com (Y.L.); bjzzzhang@163.com (Z.Z.); 2 Drilling and Production Technology Research Institute of CNPC Qinghai Oilfield, Dunhuang 736202, China; liqiangzcqh@petrochina.com.cn (Q.L.); sunzcqh@petrochina.com.cn (N.S.); cckqh@petrochina.com.cn (C.C.)

**Keywords:** co-culture, biosurfactant production, microbial enhanced oil recovery, cooperation mechanism, residual oil mobilization, field trial, microbial community

## Abstract

Microbial enhanced oil recovery (MEOR) is a promising technology for oil field extraction. This study investigated a co-culture system of *Pseudomonas aeruginosa* and *Bacillus subtilis* to increase MEOR efficacy. We analyzed bacterial growth, biosurfactant production, and crude oil emulsified performance under different inoculation ratios. Compared to single cultures, the co-culture system showed superior growth and functional expression, with an optimal inoculation ratio of 1:1. Quantitative assessments of the cell numbers and biosurfactant production during the co-culture revealed that rapid *B. subtilis* proliferation in early stages significantly stimulated *P. aeruginosa* growth. This interaction increased cell density and rhamnolipid production by 208.05% and 216.25%, respectively. The microscopic etching model displacement results demonstrated enhanced emulsification and mobilization of crude oil by the co-culture system, resulting in 94.48% recovery. A successful field application in a block-scale reservoir increased cumulative oil production by 3.25 × 10^3^ t. An analysis of microbial community structure and function in different phases revealed that after co-culture system injection, *Pseudomonas* became the dominant genus in the reservoir community, with an average abundance of 24.80%. Additionally, the abundance of biosurfactant-producing and hydrocarbon-degrading bacteria increased significantly. This research and the application of the *P. aeruginosa* and *B. subtilis* co-culture system provide novel insights and strategies for MEOR.

## 1. Introduction

Following the primary and secondary stages of oil recovery, an increasing number of oil fields enter a phase characterized by low production, in which only 20–30% of the crude oil in the reservoir is extracted [1]. A significant amount of in situ oil remains difficult to recover through natural pressure or water/gas flooding, primarily due to the adverse effects of factors such as capillary forces. To enhance crude oil recovery further, implementing tertiary oil recovery technology is essential [2]. In recent years, the main factors influencing the adoption of enhanced oil recovery (EOR) technologies by oilfields have included international oil prices, development costs, and extraction efficiency [3]. Microbial enhanced oil recovery (MEOR), which utilizes microorganisms and their metabolites to extract residual oil from reservoirs, is noted for its low cost and high efficacy, and has increasingly gained in popularity for use in oil fields [4].

The effectiveness of MEOR depends on the performance of oil recovery functional bacteria [5]. Current research focuses primarily on screening high-performance oil recovery bacteria and genetic engineering modifications of microorganisms. However, the potential for improving microbial oil displacement capacity through co-culture methods has long been ignored. Microorganisms interact through the exchange of metabolites and signaling molecules, leading to cooperative or competitive relationships that significantly influence microbial growth and functional expression [6,7]. Numerous studies have aimed to enhance microbial performance in biosurfactant production, emulsification of crude oil, and crude oil degradation through co-culture [8,9]. For example, the biosurfactant production of *Bacillus licheniformis* increased from 1.0 mg/L to 34.0 mg/L when co-cultivated with *Listeria innocua*. Similarly, co-culturing *Pseudomonas* sp. with *Listeria innocua* resulted in a substantial increase in biosurfactant production, from 13.6 mg/L to 45.2 mg/L [10]. When cultured independently, *Sarocladium* sp. and *Cryptococcus* sp. presented surface tensions of 33 mN/m and 34 mN/m, respectively, with crude oil degradation rates of 36% and 37%. However, in a co-culture system, the surface tension decreased to 31 mN/m, and the crude oil degradation rate exceeded 50% [11]. The primary mechanisms of MEOR include the production of biosurfactants and the degradation of crude oil [12]. Therefore, co-culturing microorganisms to increase MEOR effectiveness is an inspiring area for future research.

*Pseudomonas* and *Bacillus* are among the most frequently researched and applied functional bacteria for oil recovery, demonstrating significant performance, adaptability, and widespread distribution in crude oil habitats [13]. Research has shown that co-culturing these two bacterial genera results in improved performance compared to single cultures, reflected in increased growth activity, heightened cell surface hydrophobicity, and augmented emulsification activity [14]. The degradation rate of crude oil increased from 32.61% or 54.36% in single cultures of *B. licheniformis* or *P. aeruginosa* to 63.05% in the co-culture system [15]. Investigations into the cooperative mechanisms and mutualism between *Pseudomonas* and *Bacillus* during co-culture have been conducted. Co-culturing *B. licheniformis*, which produces the biosurfactant lichenin, and *P. stutzeri*, a denitrifying bacterium, showed that lichenin enhances the permeability of the *P. stutzeri* cell membrane [16]. This enhancement facilitates the transmembrane transport of nitrogen and carbon sources and improves the denitrifying ability of *P. stutzeri*. Additionally, untargeted metabolomics indicated that during the co-culture of *Pseudomonas* and *Bacillus*, *Bacillus* can provide fatty acids that promote the synthesis of rhamnolipids by *Pseudomonas*. The co-culture system demonstrated an increased capacity for biosurfactant production [17]. However, there is currently no evidence that the co-culture of *Pseudomonas* and *Bacillus* has been utilized in practical microbial enhanced oil recovery applications.

This study investigated the potential application of a co-culture system comprising *P. aeruginosa* and *B. subtilis* for enhanced oil recovery and explored the mechanisms of interaction between these two bacterial species. Initially, the growth, biosurfactant production, and emulsification properties of both single and co-culture systems were evaluated. The inoculation ratio of the strains within the co-culture system was optimized. The cell density and biosurfactant production in both the single and co-culture systems were quantified at different stages of cultivation. Additionally, the interaction mechanisms between *P. aeruginosa* and *B. subtilis* within the co-culture system were briefly elucidated. The mechanism and potential of the co-culture system for enhanced oil recovery (EOR) were analyzed through microscopic etching modeling. A block-scale enhanced recovery field trial was subsequently conducted. The production data were recorded continuously for one year after the trial, and samples were collected for microbiome analysis. This analysis aimed to investigate the evolution of species composition and potential functions within the reservoir microbial community throughout the MEOR process.

## 2. Materials and Methods

### 2.1. Bacterial Strains, Growth Conditions, and Inoculum Preparation

The two strains used in the experiment were obtained from the laboratory’s strain library and identified at the molecular level through 16S rDNA analysis, PCR amplification, and subsequent sequencing to determine their genera. The identification results indicated that *P. aeruginosa* had 99% similarity to *Pseudomonas aeruginosa* PAO1, corresponding to ATCC 47085, while *B. subtilis* had 99% similarity to *Bacillus subtilis* 168, corresponding to ATCC 23857. The *P. aeruginosa* colonies were characterized by yellowish–green coloration, round shape, opacity, regular smooth edges, and small colony size. In contrast, the colonies of *B. subtilis* were white, round, opaque, had rough and irregular edges, and were relatively large in size. Notably, the colonies of *Pseudomonas aeruginosa* and *Bacillus subtilis* used in this study presented significant morphological differences.

In this study, two different nutrient media were used. LB medium (g/L): yeast extract 5.0, tryptone 10.0, NaCl 10.0, with the pH adjusted to 7.0–7.5 using NaOH and HCl; and sucrose inorganic salt medium (g/L), comprising sucrose molasses 10, NaNO_3_ 4.0, KH_2_PO_4_ 2.0, Na_2_HPO_4_ 1.5, MgSO_4_·7H_2_O 0.5, CaCl_2_ 0.25, and 1 mL of trace element solution, including FeCl_3_·6H_2_O 0.5, CoCl_2_·6H_2_O 0.3, ZnSO_4_·7H_2_O 0.3, CuSO_4_·5H_2_O 0.2, MnSO_4_·H_2_O 0.5, Na_2_MoO_4_·2H_2_O 0.4, and H_3_BO_3_ 1.0. The pH of this medium was adjusted to 7.0–7.5 using NaOH and HCl.

Before each experiment, *P. aeruginosa* and *B. subtilis*, preserved in glycerol tubes, were inoculated into LB medium at a 2% concentration and incubated in a shaker at 140 rpm for 24 h at 37 °C. The bacterial suspension was subsequently centrifuged at 5600× *g* for 5 min, after which the supernatant was discarded. The remaining cells were resuspended in phosphate-buffered saline (PBS) at pH 7.3 to achieve an optical density (OD) of 1.0, which served as the inoculum for subsequent experiments.

### 2.2. Optimization of Inoculation Ratios and Determination of Properties in Co-Culture Systems

The experiment aimed to assess the effect of inoculum ratios on the performance of a co-culture system comprising *P. aeruginosa* and *B. subtilis*. It was conducted using triplicate 100 mL sucrose inorganic salt medium in 250 mL flasks. The flasks were inoculated with different proportions of bacterial suspensions of *Pseudomonas aeruginosa* and *Bacillus subtilis*, maintaining a total inoculum concentration of 2%. The flasks were incubated for 72 h at 140 rpm in a shaker at 37 ± 1 °C. Samples were then collected, centrifuged at 5600 rpm for 5 min, and the supernatant was separated. An equal volume of PBS was added to the supernatant and mixed thoroughly.

The bacterial density of the suspensions was determined by measuring the optical density (OD) at 600 nm using a Shimadzu UV-2600i spectrometer (Shimadzu, Shanghai, China) [18]. The surface tension, oil spreading diameter, and emulsification index of the separated supernatant were measured. The surface tension was measured using a JK99B surface tension meter (Zhongchen, Shanghai, China) at 20 °C [19]. The oil spreading diameter was determined using a modified method. For this method, 5 mL of Sudan III-stained tetradecane was spread evenly in a Petri dish containing 20 mL of deionized water. Next, 10 μL of the supernatant was added dropwise to the center of the dispersed tetradecane [20]. After stabilization, the oil spreading diameter was measured using an image analysis system. To determine the emulsification index, 4 mL of liquid paraffin was mixed with 4 mL of the supernatant at high speed for 2 min, and then allowed to stand for 24 h. The index was calculated as the ratio of the emulsion layer height to the total mixture height [21]. The analysis results established the optimal inoculation ratio of *P. aeruginosa* and *B. subtilis*, which was used in subsequent studies.

### 2.3. Determination of Cell Density and Biosurfactant Production in Culture Systems

*P. aeruginosa* and *B. subtilis* were inoculated into two separate 100 mL sucrose inorganic salt media as single-culture systems. The inocula of *P. aeruginosa* and *B. subtilis* were combined at the optimal inoculation ratio and introduced into a 200 mL sucrose inorganic salt medium, which served as the co-culture system. The inoculation ratio was consistently maintained at 2% for both culture systems. Both the single culture system and the co-culture systems were incubated in a shaker at 140 rpm and 37 °C for 5 days. At each time point, 10 mL of bacterial suspension was aspirated from each single culture and combined to form a sample for the single culture system. Similarly, 20 mL of bacterial suspension was aspirated from the co-culture system to create the sample for testing.

**Figure 1:**
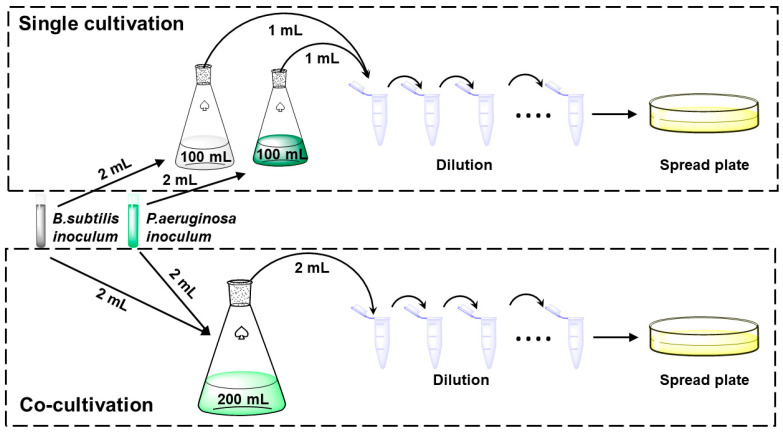
The single-culture and co-culture systems and the determination method of colony-forming unit.

The cell density and biosurfactant production were measured in both the single-culture and co-culture systems. The bacterial density of each strain was determined by counting the colony-forming units (CFUs) of *P. aeruginosa* and *B. subtilis* on LB agar plates (Figure 1) [16]. Owing to the presence of sucrose in the medium, rhamnolipids were extracted from the supernatant twice using a chloroform–ethanol (2:1) solution before being quantified by the anthrone–sulfuric acid method [22]. It is important to note that anthrone–sulfuric acid solutions are inherently unstable and require immediate analysis [23]. The cetylpyridinium chloride–bromothymol blue (CPC-BTB) colorimetric method was used for the rapid determination of surfactin production in the supernatant [24].

### 2.4. Microscopic Oil Displacement Experiments

The single-culture system and the co-culture system were injected into the microscopic etching model after water flooding. This procedure aimed to observe the movement and distribution of residual oil, calculate the crude oil recovery rate, and compare the oil displacement efficiency of the single- and co-culture systems [25].

The dimensions of the microscopic etching model used in this experiment were 45 mm × 45 mm, with a thickness of 5 mm. The pore channels in the model had diameters ranging from 20 to 40 μm, whereas the throat channels ranged from 5 to 40 μm [26]. The porosity of the model was measured at 15.88%, and the original wettability of the model was hydrophilic. The procedure for microscopic oil displacement experiments was as follows: (1) vacuum the model; (2) fully saturate it with formation water; (3) inject crude oil to saturate the entire pore space, and age it at room temperature for 48 h; (4) inject 10 pore volumes (PV) of formation water; (5) inject 2 PV of the culture system to be tested, followed by a 48 h incubation at reservoir temperature; (6) inject another 10 PV of formation water. The injection rate was maintained at 0.005 mL/min throughout the experiment [27]. After steps (4) and (6), the model was photographed and documented, and the oil recovery rate was calculated for both the single-culture and co-culture systems [28].

The oil recovery rate (%) = (initial crude oil area − residual crude oil area)/initial crude oil area × 100%

The emulsified oil droplets were collected after the displacement experiments. These droplets were examined using a microscope (Olympus BX51, Tokyo, Japan) at room temperature. The particle size distributions of the oil droplets were analyzed using ImageJ software (https://imagej.net/ij/) [29].

### 2.5. Oilfield Geochemical Features and MEOR Strategy

The MEOR field trial was conducted in the CHE 362 Block, located in Chepaizi Oilfield, Xinjiang, China, in the southern part of the Hongche fault zone of the western uplift of the Junggar Basin (Figure S1). The studied block included 18 injection wells and 36 production wells, which were developed with a well spacing of 250 m, using both anti-seven-point and anti-five-point injection and recovery well patterns. The wells underwent three development stages: natural energy extraction, water flooding, and increased water content. The reservoir’s average depth is 1682.6 m, with a temperature of 47.5 °C and a pressure of 17.53 MPa. It has an average porosity of 19.08% and an average permeability of 2.79 mD, indicating a medium-porosity, low-permeability reservoir. After a prolonged period of water injection development, the average water content of the reservoir reached 68.5%. The density of the crude oil was measured at 0.923 g/cm^3^ at 50 °C, with a viscosity of 208.40 mPa·s. An analysis of the trial block reservoir environment (Table S1) revealed significant differences between the reservoir conditions and the laboratory cultivation conditions. However, the growth conditions in both the laboratory and field experiments fall within the optimal growth range for the two microorganisms used in this study (Figure S2). Therefore, the differences in growth conditions do not significantly affect microbial growth performance.

In this study, *P. aeruginosa* and *B. subtilis* were co-cultured in a fermenter at a 1:1 ratio for 48 h. The resulting fermentation solution was mixed with a nutrient solution and injected into the reservoir through the injection pipeline, driven by the injected water. The injection period lasted from 16 August 2022, to 30 November 2022, during which 40 m^3^ of microbial fermentation solution and 214 m^3^ of nutrient solution were injected. The comprehensive MEOR field trial spanned from February 2022 to June 2023. For sampling, 10 production wells were selected, and samples were collected at three stages: pre-injection (30 May 2022), injection (10 November 2022), and post-injection (30 March 2023). During sampling, the sampling valve was opened, and the water samples were allowed to flow for 5 min to stabilize, minimizing the influence of the pipeline equipment and residual liquid. After sampling, the samples were placed in 5 L sterilized polyethylene containers, sealed with plastic film and threaded caps to prevent contamination. The samples were then transported to the laboratory within 72 h and stored at 4 °C for further analysis.

### 2.6. High-Throughput Sequencing and Microbial Community Analysis

The microbial genome was extracted using the CTAB method. Universal primers (515F: GTGCCAGCMGCCGCGGTAA and 806R: GGACTACHVGGGGTWTCTAAT) were selected to amplify the V4 region of the 16S rDNA gene. The amplified gene was then sequenced on the Illumina platform using the NovaSeq 6000 system. The sequences were grouped into operational taxonomic units (OTUs) with a 97% identity threshold. Species annotation was performed using the Silva 138.1 database with the RDP taxonomic annotation algorithm. Multiple sequence comparisons were carried out using MUSCLE v3 software, and a phylogenetic tree was constructed [30]. The sample with the least amount of data was used for homogenization, and the processed data were used for further analysis. Functional prediction on the basis of high-throughput sequencing results was performed using the FAPROTAX (Functional Annotation of Prokaryotic Taxa) and PICRUSt2 (Phylogenetic Investigation of Communities by Reconstruction of Unobserved States) databases [31,32].

### 2.7. Statistical Analyses

All the experimental results are presented as the means ± standard deviation (SD) based on three parallel experiments. Significant differences in all statistical results were determined by independent-samples *t*-test and one way ANOVA with Duncan’s multiple range test (*p*-values < 0.05*, 0.01**, 0.001***). All significant differences in analyses were determined using IBM SPSS Statistics 27.0.

## 3. Results and Discussion

### 3.1. Growth, Biosurfactant Production, and Emulsification Properties in Co-Culture Systems with Different Inoculum Ratios

**Figure 2:**
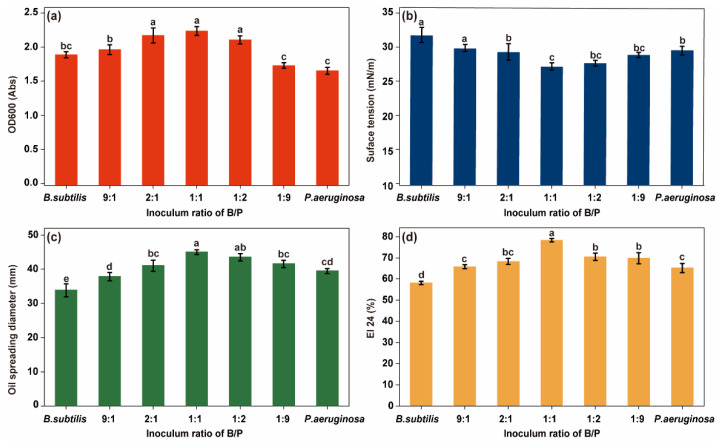
(**a**) The OD600 values; (**b**) Surface tension; (**c**) Oil spreading diameter: (**d**) Emulsification index in culture systems with different inoculum ratios. Letters on the graph indicate significant differences between the data.

Microbial growth performance and functional expression capacity are key factors influencing oil recovery performance. The growth, surfactant production, and emulsification by microorganisms can be quantified to indirectly assess the oil recovery performance of these organisms. Microbial growth was evaluated by measuring the optical density (OD) of the bacterial solution at 600 nm (Figure 2a). To quantify the biosurfactant production, both the surface tension and the oil spreading diameter were measured (Figure 2b,c). The emulsification index of the bacterial solution was determined to assess its ability to emulsify crude oil (Figure 2d). After 48 h of incubation, the OD 600 of *Bacillus subtilis* was greater than that of *Pseudomonas aeruginosa*, yet the degrees of reduction in the surface tension of the bacterial fluid, the diameter of the oil drainage circle, and the EI 24 were smaller. This result indicates that, under the experimental conditions, *B. subtilis* exhibits superior growth performance but weaker oil-recovery-related functions than *P. aeruginosa*. Compared to their single cultures, the co-culture of *P. aeruginosa* and *B. subtilis* at different inoculum ratios showed an increase in OD 600, oil spreading diameter, and emulsification index in the bacterial solution, while the surface tension of the bacterial solution significantly decreased (Table S2). These results indicate that bacterial growth, biosurfactant production, and emulsification were significantly improved in the co-culture of *P. aeruginosa* and *B. subtilis* compared to their individual cultures. At inoculum ratios of B/P = 2:1, 1:1, and 1:2, the OD 600 was significantly higher than in the other groups. When the inoculum ratio of B/P was 1:1, the bacterial solution showed the significantly higher oil spreading diameter and emulsification index, with significantly lower surface tension (*p* < 0.05). These results indicated that the ability to produce biosurfactants and emulsify crude oil exhibited a positive correlation with bacterial growth across all the inoculation ratios of the co-culture system [33,34]. The co-culture system with a 1:1 inoculum ratio of *P. aeruginosa* and *B. subtilis* exhibited the highest cell density (OD 600: 2.21), the highest biosurfactant production (surface tension = 27.08 mN/m, oil spreading diameter = 45.00 mm), and the greatest emulsification capacity (EI 24 = 78.17%). The most significant performance improvement was observed at the 1:1 inoculum ratio, which was used for further investigations.

### 3.2. Effects of the Co-Culture System on Microbial Growth and Biosurfactant Production

**Figure 3:**
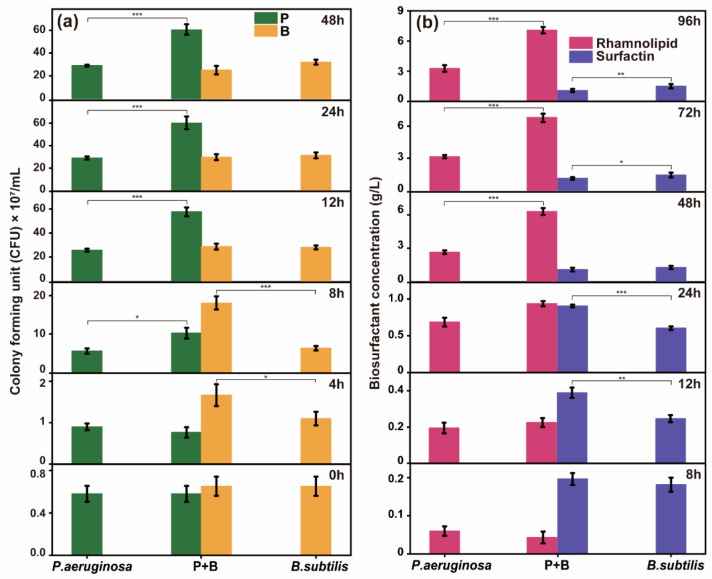
(**a**) Cell numbers of *P. aeruginosa* and *B. subtilis*; (**b**) Rhamnolipid and surfactin production in single culture and co-culture. ***, *p* < 0.001; **, *p* < 0.01; *, *p* < 0.05.

Studying the mechanisms of microbial interactions can better realize microbial functional expression and enhance oil recovery performance. To investigate the interaction between *P. aeruginosa* and *B. subtilis* during co-culture, we measured the cell density and biosurfactant production in both single- and co-culture systems, and the impact of co-culture on the growth and metabolic rates of both *P. aeruginosa* and *B. subtilis* were analyzed. After 48 h of incubation, the microbial growth plateaued, with minimal changes in cell density, and the biosurfactant production stabilized after 96 h. Thus, the cell density and biosurfactant production were measured during these two growth phases (Figure 3).

In the early culture stages, the *B. subtilis* in the co-culture showed significantly higher cell density than that in the single culture, whereas *P. aeruginosa* showed similar cell densities under both conditions. Notably, at 8 h, the cell density of the *B. subtilis* in the co-culture was 210.42% of that in the single culture, which was a statistically significant difference (*p* < 0.001). These findings suggest that the co-culture initially stimulated *B. subtilis*, boosting its growth. However, as the incubation period continued, the difference in *B. subtilis* cell density between the co-culture and single cultures gradually decreased. By 12 h, the *B. subtilis* cell density in the co-culture had decreased to 106.86% of that in the single culture, and by 48 h, it had further declined to 78.13%. Moreover, the cell density of the *P. aeruginosa* in the co-culture was significantly higher than that in the single culture (*p* < 0.001), reaching 208.05% by the conclusion of the experiment (Figure 3a). These results indicate that the growth rate of *P. aeruginosa* in the co-culture surpassed that in the single culture during later incubation stages. A similar trend was observed in biosurfactant production. During the early metabolic stage, the surfactin production in the co-culture was significantly higher than in the single culture, reaching 150.00% at 24 h. The difference in rhamnolipid production between the co-culture and single cultures was minimal. However, in the later stages of metabolism, the difference in surfactin production between the co-culture and the single culture gradually decreased, whereas the rhamnolipid production in the co-culture was significantly higher than that in the single culture, reaching 216.25% by the end of the experiment (Figure 3b).

The growth and metabolic rates of both *B. subtilis* and *P. aeruginosa* were significantly enhanced in the co-culture system compared to their respective single cultures. *B. subtilis* showed a greater ability to utilize the initial metabolic substrate (sucrose molasses) than *P. aeruginosa* [35,36,37,38], an observation that is confirmed in the sucrose metabolism pathway diagram of *P. aeruginosa* and *B. subtilis* (Figure S3). In the early stages of the co-culture, *B. subtilis* occupied more available nutrients and growth space, which increased its growth rate, leading to increased cell density and biosurfactant production [39]. Additionally, *B. subtilis* exhibited a population effect, wherein cell density positively correlated with *ComXA* activity, a regulator of the surfactin-producing gene, thereby further enhancing surfactin production [40]. The rapid proliferation of *B. subtilis* is more effective at decomposing molasses into monosaccharide carbon sources, such as glucose and fructose. The analysis of the growth performance of *P. aeruginosa* and *B. subtilis* in media with different sugar carbon sources (Figure S4) demonstrated that *P. aeruginosa* utilizes monosaccharide carbon sources more effectively, thus stimulating the growth activity of *P. aeruginosa*. This process stimulated the growth rate of *P. aeruginosa*, which displayed a strong capacity to utilize these monosaccharide carbon sources [41,42]. The surfactin produced by *B. subtilis* also enhanced the permeability of the cell membranes of *P. aeruginosa*, facilitating nutrient uptake efficiency [16]. Consequently, this interaction increased the growth rate of *P. aeruginosa*, characterized by a rapid increase in cell density and rhamnolipid production within the co-culture system. As the bacterial density increased, the interaction between *B. subtilis* and *P. aeruginosa* shifted from mutualistic to inhibitory competition. The coding factor H2-T6SS and the metabolite chlorophyll produced by *P. aeruginosa* inhibited the growth rate of *B. subtilis* [43,44]. Conversely, the surfactant present in the co-culture system enhanced the mobility of both *B. subtilis* and *P. aeruginosa* colonies, intensifying competitive dynamics [45]. This ultimately reduced the growth rate of *B. subtilis* and ceased cell density increase and surfactin production in the later stages of the co-culture. In contrast, *P. aeruginosa* exhibited heightened activity, resulting in a significant increase in both cell density and rhamnolipid production compared with the single cultures.

### 3.3. Enhanced oil recovery potential of the Co-Culture System 

Microscopic oil displacement experiments can provide a more intuitive evaluation of the oil displacement performance of the flooding system and help to explain the displacement mechanisms. To evaluate the oil displacement efficacy of a co-culture system consisting of *P. aeruginosa* and *B. subtilis*, laboratory oil flooding experiments were conducted using a microscopic etching model. When the injected fluid was introduced into the model from the injection end, it initially entered the low-resistance pore, displacing the crude oil toward the outlet end. Once the fluid broke through at the outlet end, the middle channel area formed between the injection and outlet ends. Subsequent injections of fluid preferentially traversed this middle channel area, displacing most of the crude oil within it. The region surrounding the middle channel area, which was within the sweep scope of the injected fluid but showed low displacement efficiency, was defined as the excessive area. The area beyond the reach of the injection fluid was referred to as the boundary dead-end area [25].

**Figure 4:**
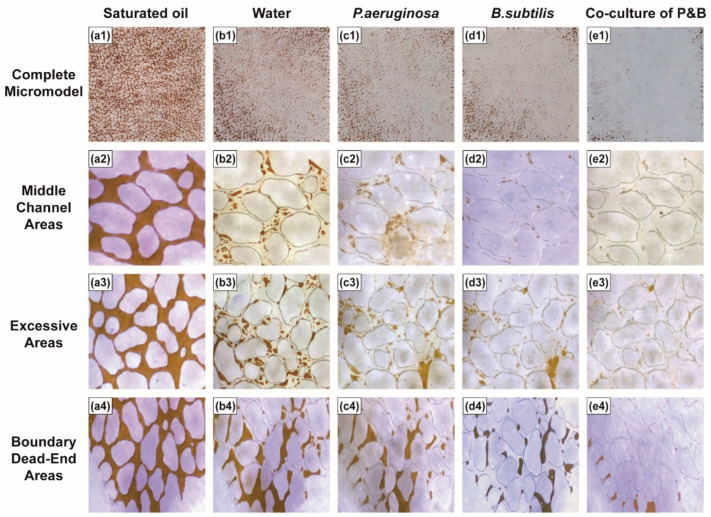
Characteristics of residual oil distribution in different regions in the microscopic etching model. (**a1**–**a4**) Saturated oil; (**b1**–**b4**) Water flooding; (**c1**–**c4**) *P. aeruginosa* flooding; (**d1**–**d4**) *B. subtilis* flooding; (**e1**–**e4**) Co-culture of P and B.

Following water flooding, a significant portion of the crude oil within the model was displaced, resulting in an oil recovery rate of 61.11% (Figure 4(b1)). Residual oil in the middle channel area consists of droplets, filamentous, and columnar oil; in the excessive area, residual oil is found in the form of droplets, filamentous, columnar, porous, and cluster oil; and in the boundary dead-end area, residual oil is mainly found as clusters, droplets, and filamentous forms [29]. Upon the injection of a microbial oil displacement system, a considerable quantity of residual oil was removed, leading to varying degrees of improvement in the oil recovery rate. Specifically, the application of single-culture systems of *P. aeruginosa* and *B. subtilis* resulted in oil recovery rates of 71.90% (Figure 4(c1)) and 83.22% (Figure 4(d1)), respectively. The quantity and size of residual oil droplets and filamentous structures decreased significantly, while the quantity of columnar and porous residual oil approached zero, and the size of cluster-like residual oil decreased drastically. The reduction in droplets and filamentous residual oil was mainly due to the high concentration of biosurfactants in the microbial enhanced oil recovery (MEOR) system. Biosurfactants reduce the interfacial tension between oil and water, weakening the strength of the oil–water interface film and increasing the shear force of the displacement fluid on the oil film, which causes the droplets and filamentous structures to detach from the rock surface. Additionally, the injection of the microbial system alters the rheological properties of the displacement fluid, disrupting the balance of displacement forces on columnar and porous residual oil in multiple directions. This results in the application of a sufficient force to displace these structures. Furthermore, the MEOR system also changes the flow ratio of the displacement fluid, leading to increased swept volume. Residual oil in regions not previously swept by water flooding is displaced, causing a significant amount of cluster residual oil to be removed. After injecting the co-culture system of *P. aeruginosa* and *B. subtilis* into the water-flooded microscopic system and performing secondary water flooding, the oil recovery rate was 94.48% (Figure 4(e1)). Most of the residual oil was displaced, and it was difficult to observe droplets and filamentous residual oil in the main flow channel, indicating that the co-culture system significantly enhances the oil displacement efficiency. In the excessive area after the water flooding, the distribution and state of residual oil in the pore throat channels, following displacement by the co-culture system, resembled those in the middle channel area, and can be classified as part of the middle channel area. These results suggest that the co-culture system increases the swept volume, and the reduction in the distribution range of cluster-like residual oil in hard-to-sweep regions is due to the increased swept volume and displacement capacity of the system. Furthermore, there was a significant reduction in the residual oil within the excessive region. The experimental findings suggest that various microbial oil displacement systems can effectively improve oil recovery. The co-culture system of *P. aeruginosa* and *B. subtilis* demonstrated a superior oil recovery rate in the excessive and boundary dead-end areas. These results indicate that the co-culture system exhibits greater oil displacement efficiency and sweep efficiency compared to single-culture systems.

**Figure 5:**
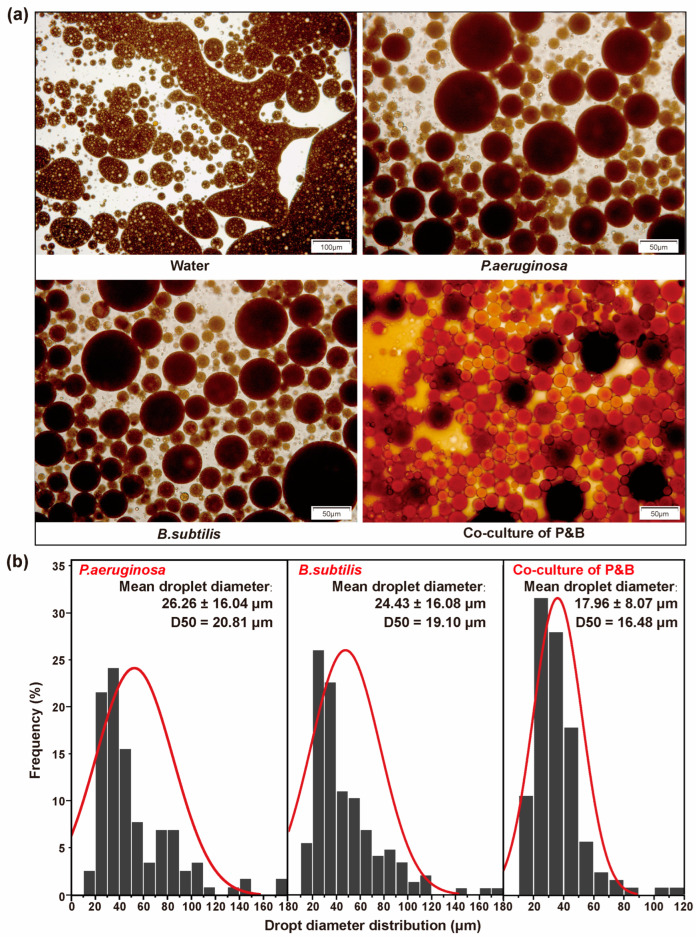
Emulsion following various fluid displacements. (**a**) Microscopic image of emulsified oil droplets; (**b**) Particle size distribution of the oil droplets. The red curve is the size composition distribution curve.

To investigate the effects of crude oil emulsification by various microbial oil displacement fluids, the recovered fluid after displacement was examined microscopically, and the mean droplet diameter of the oil droplets in the recovered fluid was calculated. The oil phase in the product liquid resulting from water flooding was observed to exist in the form of continuous flakes, indicating a low degree of oil/water emulsification. In contrast, following the injection of different microbial oil displacement fluids, the oil phase in the produced liquid was dispersed in the water phase as droplets, forming an oil/water emulsion (Figure 5a). The mean droplet diameter of the oil droplets displaced by the co-culture system (17.96 ± 8.07 μm) was significantly smaller than that of the *P. aeruginosa* (24.43 ± 16.08 μm) and *B. subtilis* (26.26 ± 16.04 μm) in the single cultures (Figure 5b). These findings indicate that co-culture significantly enhances the ability of microorganisms to emulsify crude oil during the displacement process. As the emulsification of the crude oil increased, the dispersion of the oil phase within the water phase also increased, leading to a reduction in the volume of the oil droplets [46]. This reduction further decreased the adsorption of crude oil on the formation surface and the capillary forces within the reservoir pores, thereby facilitating the expulsion of the oil phase. Following the formation of an emulsion, the fluidity ratio between the water and crude oil changed, resulting in improvements in the displacement efficiency and sweep efficiency of the microbial oil displacement fluid [47]. The co-culture system demonstrated a significantly enhanced capacity to emulsify crude oil, which contributed to its superior sweep and displacement efficiency.

### 3.4. Production Performance of the MEOR Field Trial Blocks

**Figure 6:**
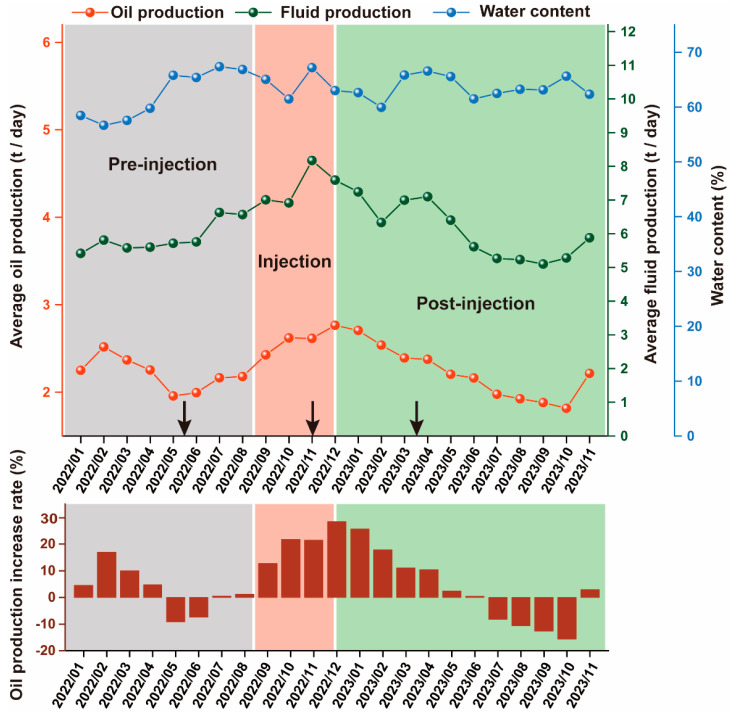
Production performance and oil production increase rates in the field trial. The arrows represent sampling dates. Gray coding indicates the pre-injection phase, red coding indicates the injection phase, and green coding indicates the post-injection phase. The pre-injection, injection, and post-injection phases together constitute the complete MEOR experimental cycle.

Statistical analyses of field production data during the MEOR experimental cycle can provide a clearer evaluation of the MEOR application effect and support the development of MEOR. During the pre-injection phase, crude oil production gradually stabilized after a continuous decrease over four months. The average daily oil production was 2.15 t/d, with a water content of 63.78%. In the injection phase, oil production continued to rise, and the production curve maintained a clear upward trend. The average daily oil production increased to 2.48 t/d, and the oil production curve showed an upward trend. The water content increased to 64.10%, and the rate of increase in water content exhibited a deceleration. The oil production increase rate was 15.35%. Between August 2022 and June 2023, the cumulative increase in production was 3.25 × 10^3^ t, indicating a substantial improvement in oil recovery (Figure 6). Six months after the MEOR was stopped, the oil production levels returned to those observed before the MEOR was implemented, suggesting that the increase in oil production was primarily due to the MEOR application in the field. The change in oil production was strongly correlated with the intensity of MEOR interference on the trial block. Following the injection of microbial flooding agents and nutrients, the interference from MEOR in the well gradually increased, and production rose accordingly. After the injection phase, nutrients in the reservoir were gradually depleted, and the activity of the injected exogenous oil-producing microorganisms and activated endogenous oil-producing microorganisms gradually decreased. As a result, the interference from MEOR decreased, leading to a continuous decline in oil production, eventually falling to the same level as or below the production levels observed during the pre-injection phase. To maintain the enhanced production efficiency of the oil field, continuous microbial intervention is necessary [6].

At the end of this MEOR field trial, the economic benefits were further calculated and a cost reduction improvement method was briefly proposed. The MEOR field trial resulted in an additional 3250 tons of crude oil. Based on the 2023 average crude oil price (USD 82.04 per barrel), the estimated value of this production increase is approximately USD 1.85 million. The total MEOR field trial cost was USD 150,000, including bacterial solution, nutrients, equipment rental, labor, transportation, and post-trial testing. These results demonstrate the high economic benefits of this MEOR field trial. In the laboratory study, distilled water was used to prepare a sucrose inorganic salt medium. For the field trial, the medium was prepared using injection water, which already contained essential trace elements (such as Ca, Mg, Fe, and Mn) required by the microorganisms. Only a carbon source, nitrogen source, and phosphorus source needed to be added, reducing the overall field trial cost of MEOR application.

### 3.5. Bacterial Composition of the Reservoir Community in Oil Wells Across Various Phases of MEOR

**Figure 7:**
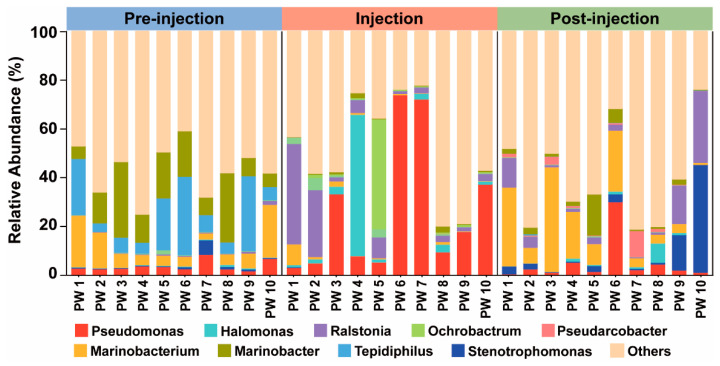
The relative abundances of bacterial orders in the representative oil well during different MEOR phases. Only the top 10 orders are visualized.

A total of 30 samples from oil wells were collected during the three phases of the MEOR field trial. The reservoir microbial community was analyzed at the genus level to determine its compositional structure during the pre-injection, injection, and post-injection phases (Figure 7). The results indicated that following microbial intervention, the bacterial distribution within the reservoir communities of different oil wells underwent significant changes. Although the specific trends of these alterations were not uniform, they exhibited certain similarities. In the pre-injection phase, the relative abundance of *Pseudomonas* was lower (4.45%), whereas the relative abundances of *Marinobacterium* (8.82%), *Marinobacter* (14.65%), and *Tepidiphilus* (13.81%) were higher in the reservoir communities. *Marinobacterium*, *Marinobacter*, and *Tepidiphilus* have been less extensively studied in the context of crude oil degradation and biosurfactant production, suggesting that these genera may have limited potential to influence the quality of crude oil or to enhance its mobility. During the injection phase, the relative abundance of *Pseudomonas* in the reservoir communities increased significantly, with an average relative abundance of 24.80% across the oil wells and a maximum of 74.07%. The relative abundance of *Pseudomonas* in the injected exogenous bacteria significantly increased, while the relative abundance of *Bacillus* showed no noticeable change, which is consistent with the results of laboratory co-culture studies. After 24 h of co-culture on the overground, the injected strains entered a phase in which the growth performance of *Bacillus* weakened, while that of *Pseudomonas* improved. *Pseudomonas* exhibited stronger growth and competitiveness than *Bacillus*, leading to a significant increase in the relative abundance of *Pseudomonas* in the reservoir. It was also observed that, in the initial state of the reservoir microbial community, *Bacillus* had a relatively low abundance, while *Pseudomonas* had a higher relative abundance, suggesting the better adaptability of *Pseudomonas* to the reservoir environment. This may explain why the relative abundance of *Pseudomonas* increased significantly while that of *Bacillus* did not. Concurrently, the relative abundances of *Ralstonia* (9.70%), *Halomonas* (7.06%), and *Ochrobactrum* (5.08%) also increased markedly, establishing themselves as the dominant genera within the microbial community. The significant increase in the relative abundance of *Pseudomonas* can be attributed to the synergistic effects of exogenous bacteria injected and the activation of indigenous bacterial populations. In contrast, the increase in the relative abundance of *Ralstonia*, *Halomonas*, and *Ochrobactrum* appears to be primarily a result of the activation of indigenous bacteria. The predominant genera within the microbial community of the reservoir included *Pseudomonas*, *Ralstonia*, *Halomonas*, and *Ochrobactrum*. These genera are frequently utilized as biosurfactant-producing bacteria or as agents for the degradation of petroleum hydrocarbons in the field of MEOR and in the remediation of oily pollutants [48,49,50]. Their remarkable ability to enhance the quality and fluidity of crude oil has led to a significant increase in oil production during the injection phase. Following the completion of MEOR, the relative abundance of *Pseudomonas* (5.15%), *Ralstonia* (7.05%), *Halomonas* (1.26%), and *Ochrobactrum* (0%) within the reservoir community exhibited more pronounced declines. In contrast, the relative abundance of genera with diminished oil recovery capacity, including *Marinobacterium* (14.60%), *Sulfurospirillum* (4.85%), and *Marinobacter* (3.32%), increased significantly. This reduction in the oil recovery capacity of the microbial community led to a decrease in oil production.

### 3.6. Potential Functions of the Reservoir Community in Oil Wells Across Various Phases of MEOR

**Figure 8:**
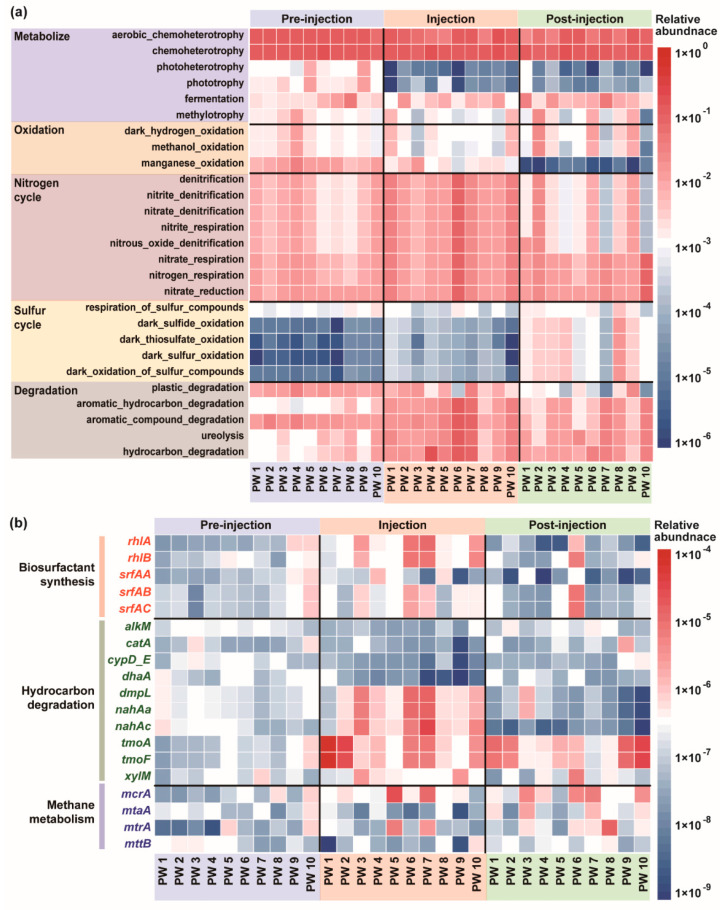
(**a**) The potential functions were predicted by FAPRTAX. (**b**) Functional genes were predicted by PICRUSt2. The data were normalized before visualization.

A comparative analysis of the microbial communities in the reservoirs at different stages of MEOR, using the FAPROTAX database, enabled the prediction of their biogeochemical functions, including elemental cycling (carbon, nitrogen, and sulfur), fermentation, and degradation processes (Figure 8a). During the injection phase, the microbial communities exhibited enhanced capabilities in nitrogen and sulfur cycling, such as denitrification, respiration, and the reduction in nitrogen-containing compounds, along with sulfur and sulfide oxidation. Additionally, there was a notable increase in the degradation of hydrocarbons and aromatic compounds. In the post-injection phase, the effects of MEOR decreased, leading to a reduced capacity for nitrogen cycling, an increased role in sulfur cycling, and a further decline in hydrocarbon degradation [51].

The roles of microorganisms in oil recovery can be categorized into three primary functions: biosurfactant synthesis, methane metabolism, and hydrocarbon degradation [52,53]. A comparative analysis of the reservoir microbial communities at different phases of oil extraction, using the PICRUSt2 database, the annotation of marker genes related to oil recovery, and the tracking of changes in their relative abundance, provides valuable insights into the impact of MEOR on the oil displacement function of these communities (Figure 8b). The results revealed that the activities of the rhamnolipid-synthesizing genes *rhlA* and *rhlB*, and the surfactin-synthesizing genes *srfAA*, *srfAB*, and *srfAC*, increased following microbial intervention, but declined significantly during the post-injection phase [54,55]. This suggests that the MEOR trial enhanced the biosurfactant synthesis capacity of the reservoir microbial community. During the injection phase, the activities of the hydrocarbon-degrading genes *alkM*, *catA*, *cypD_E*, and *dhaA* decreased, whereas those of *dmpL*, *nahAa*, *nahAc*, *tmoA*, *tmoF*, and *xylM* increased [56]. This shift is likely to have been due to microbial intervention, which altered the species distribution in the reservoir, resulting in a significant replacement of hydrocarbon-degrading bacteria. Compared with those in the pre-injection phase, the hydrocarbon degradation gene activities were significantly higher during the injection phase, followed by a decline in the post-injection phase. These findings indicate that microbial intervention positively influences the hydrocarbon degradation capacity of microbial communities. The microbial degradation of hydrocarbons can reduce impurities in crude oil, decrease viscosity, and enhance fluidity, thus facilitating extraction. Changes in the activities of the methane metabolism genes *mcrA*, *mataA*, *mtrA*, and *mttB* suggest that MEOR also improves the methane metabolism capacity of reservoir microbial communities, which benefits crude oil recovery [57]. A comprehensive analysis of the functional genes revealed that MEOR increases the ability of reservoir microbial communities to perform biosurfactant synthesis, degrade hydrocarbons, and metabolize methane, leading to a significant increase in oil recovery.

## 4. Conclusions

In this study, co-culture experiments with *Pseudomonas aeruginosa* and *Bacillus subtilis* were conducted. Compared with those in single cultures, microbial growth, biosurfactant production, and emulsification properties were more pronounced in the co-culture system. During the co-culture process, *B. subtilis* metabolized sucrose molasses into monosaccharides, providing a beneficial carbon source for *P. aeruginosa*. Additionally, the biosurfactants produced by *B. subtilis* improved the permeability of *P. aeruginosa* cell membranes, accelerating nutrient transport. This ultimately increased both cell density and the rhamnolipid production of *P. aeruginosa*. The results from the microscopic etching modeling of displacement experiments indicated that the co-culture system exhibited significantly greater potential for oil recovery, achieving a recovery rate of 94.48%. In contrast, the single cultures of *B. subtilis* and *P. aeruginosa* presented lower recovery rates of 83.22% and 71.90%, respectively. A field trial for block-scale MEOR resulted in a cumulative production increase of 3.25 × 10^3^ t. After the co-culture system was injected into the reservoir, the relative abundance of *Pseudomonas* increased significantly, peaking at 74.07%, making it the dominant genus in the reservoir community. Additionally, the microbial function predictions suggested that the ability of microbial communities to produce biosurfactants and degrade crude oil was enhanced, aligning with oil recovery enhancement. This study demonstrated the potential of co-cultures to improve microbial oil recovery capabilities. However, further research is necessary to elucidate the interactions among oil recovery functional bacteria during the co-culture process.

## Data Availability

The original contributions presented in the study are included in the article/supplementary material, further inquiries can be directed to the corresponding author.

## Supplementary Materials

The following supporting information can be downloaded at: https://www.mdpi.com/article/10.3390/microorganisms12112343/s1. Table S1. Characterization of water geochemical property and reservoir temperature from ten production wells. Table S2. Growth, biosurfactant production and emulsification properties in culture systems with different inoculum ratios. Figure S1. The distribution of the oil wells. The distance between the oil wells is roughly same. Figure S2. Growth performance of (a) *B. subtilis*, (b) *P. aeruginosa*, (c) co-culture system in sucrose inorganic salt medium of different pH. (d) *B. subtilis* (e) *P. aeruginosa*, (f) co-culture system in sucrose inorganic salt medium at different temperatures. (g) *B. subtilis*, (h) *P. aeruginosa*, (i) co-culture system in sucrose inorganic salt medium with different salinity. Figure S3. Metabolism of sucrose by *B. subtilis* and *P. aeruginosa* co-culture system to produce biosurfactants. Figure S4. Growth activity of *B. subtilis* and *P. aeruginosa* in separate and co-culture systems in different carbon sources.
