# Supplementary material for: Enhanced Oil Recovery in a Co-Culture System of Pseudomonas aeruginosa and Bacillus subtilis
Source: Microorganisms. 2024 Nov 16;12(11):2343. doi: 10.3390/microorganisms12112343 (PMC11596543; doi:10.3390/microorganisms12112343)
Supplement: Supplementary file 1 [file microorganisms-12-02343-s001.zip › microorganisms-3302631-supplementary.pdf]

Table S1. Characterization of water geochemical property and reservoir temperature from ten production wells.

Period	Well ID	**Reservoir temperature**	**pH**	**Salinity (mg/L)**	**Major elements composition (mg/L)**								
					Na	K	Ca	Mg	Si	S	Cl	TP	TN
Pre-injection	PW 1	47.25	7.41	17117	3706.19	342.59	2134.41	1293.35	21.52	7.09	15255.81	46.46	67.25
Pre-injection	PW 2	45.06	7.61	18375	3907.26	432.07	2216.91	506.41	33.91	8.02	10854.74	60.45	50.76
Pre-injection	PW 3	48.30	7.21	18025	3910.93	304.08	2334.64	589.60	37.85	14.24	10495.76	94.45	71.76
Pre-injection	PW 4	46.81	7.25	14675	5108.39	432.78	796.59	469.32	84.82	9.69	7722.98	25.54	56.08
Pre-injection	PW 5	49.79	7.66	14243	4840.72	263.22	396.90	511.48	33.61	5.13	8067.66	79.91	69.56
Pre-injection	PW 6	46.23	7.72	14370	5297.51	172.94	186.43	397.84	69.75	7.89	7144.43	40.04	74.80
Pre-injection	PW 7	48.51	7.48	16249	3818.54	309.97	2092.34	479.97	21.70	4.45	9269.50	42.39	57.23
Pre-injection	PW 8	46.06	7.73	14352	4535.51	259.45	80.08	510.27	29.77	8.89	8714.84	121.19	78.13
Pre-injection	PW 9	47.24	7.61	17329	3366.43	214.4	2121.15	723.89	22.54	4.58	10403.47	47.97	57.45
Pre-injection	PW 10	49.11	7.80	16591	5523.05	211.54	1405.14	963.94	30.86	3.98	10325.74	58.90	58.89
Injection	PW 1	47.25	7.46	16923	4488.45	504.01	2138.19	1010.53	44.42	3.22	13945.36	25.79	91.03
Injection	PW 2	45.06	7.51	16653	2746.34	267.66	2680.58	397.96	27.35	7.44	10464.68	15.89	83.75
Injection	PW 3	48.30	7.56	19343	5355.09	318.35	1707.12	703.04	41.43	7.87	11044.62	23.86	56.00
Injection	PW 4	46.81	7.52	13204	4936.67	232.04	724.27	456.29	84.67	4.39	6721.68	32.55	63.42
Injection	PW 5	49.79	7.66	15679	6863.10	317.81	138.07	546.64	204.46	23.66	7296.95	39.90	79.81
Injection	PW 6	46.23	7.47	13124	4316.53	117.93	592.82	459.78	37.96	1.34	7340.52	37.46	48.29
Injection	PW 7	48.51	7.26	17710	4890.25	202.29	1877.04	373.12	18.96	1.94	10294.29	10.77	39.35
Injection	PW 8	46.06	7.33	16820	7312.30	229.16	298.03	861.85	63.86	2.82	10383.23	21.60	55.60
Injection	PW 9	47.24	7.66	15732	6626.96	186.86	95.38	454.31	52.01	2.67	8278.72	20.76	79.81
Injection	PW 10	49.11	7.71	16552	6604.69	253.76	1235.78	790.70	49.33	7.43	10527.19	23.18	77.78
Post-injection	PW 1	47.25	7.33	16321	3964.84	241.01	1590.77	1001.16	18.75	1.29	9460.22	11.83	38.57
Post-injection	PW 2	45.06	7.34	17280	3566.88	327.88	2379.22	359.18	105.96	13.19	10487.37	16.04	55.86

Period	Well ID	**Reservoir temperature**	**pH**	**Salinity (mg/L)**	**Major elements composition (mg/L)**								
					Na	K	Ca	Mg	Si	S	Cl	TP	TN
Post-injection	PW 3	48.30	7.38	17282	4649.09	296.23	1692.27	584.66	30.75	4.13	9986.32	17.44	77.35
Post-injection	PW 4	46.81	7.25	14657	5463.19	203.69	509.40	462.05	72.59	8.05	7896.85	23.22	65.08
Post-injection	PW 5	49.79	7.68	13896	6500.30	178.83	183.85	305.98	120.6	6.76	6540.21	23.55	46.29
Post-injection	PW 6	46.23	7.58	13612	4164.52	145.73	317.63	432.41	27.94	2.58	8474.32	16.12	57.34
Post-injection	PW 7	48.51	7.26	16108	3310.30	167.69	2390.07	388.54	42.47	3.99	9656.99	30.13	62.99
Post-injection	PW 8	46.06	7.32	15729	5159.55	172.79	94.62	316.52	100.79	3.09	9826.36	29.05	52.52
Post-injection	PW 9	47.24	7.27	16884	3611.98	409.37	1935.65	633.72	50.50	4.13	9887.65	26.47	54.89
Post-injection	PW 10	49.11	7.80	15070	4853.63	189.42	1390.01	436.92	30.12	1.90	8133.80	16.01	61.68

Table S2. Growth, biosurfactant production and emulsification properties in culture systems with different inoculum ratios

Inoculation ratio (B/P)	OD600 (Abs)	Surface tension (mN/m)	Oil spreading diameter (mm)	EI 24 (%)
B.subtilis	1.87 ±0.04	31.58 ± 1.09	33.83 ± 1.93	58.00 ± 0.82
9:1	1.95 ± 0.07	29.72 ± 0.49	37.83 ± 1.18	65.67 ± 0.94
2:1	2.15 ± 0.11	29.18 ± 1.19	41.00 ± 1.63	68.17 ± 1.43
1:1	2.22 ± 0.06	27.08 ± 0.53	45.00 ± 0.71	78.17 ± 0.85
1:2	2.09 ± 0.06	27.60 ± 0.41	43.50 ± 1.08	70.33 ± 1.70
1:9	1.72 ± 0.04	28.79 ± 0.38	41.50 ± 1.08	69.67 ± 2.62
P.aeruginosa	1.64 ± 0.05	29.44 ± 0.62	39.50 ± 0.71	65.00 ± 2.16

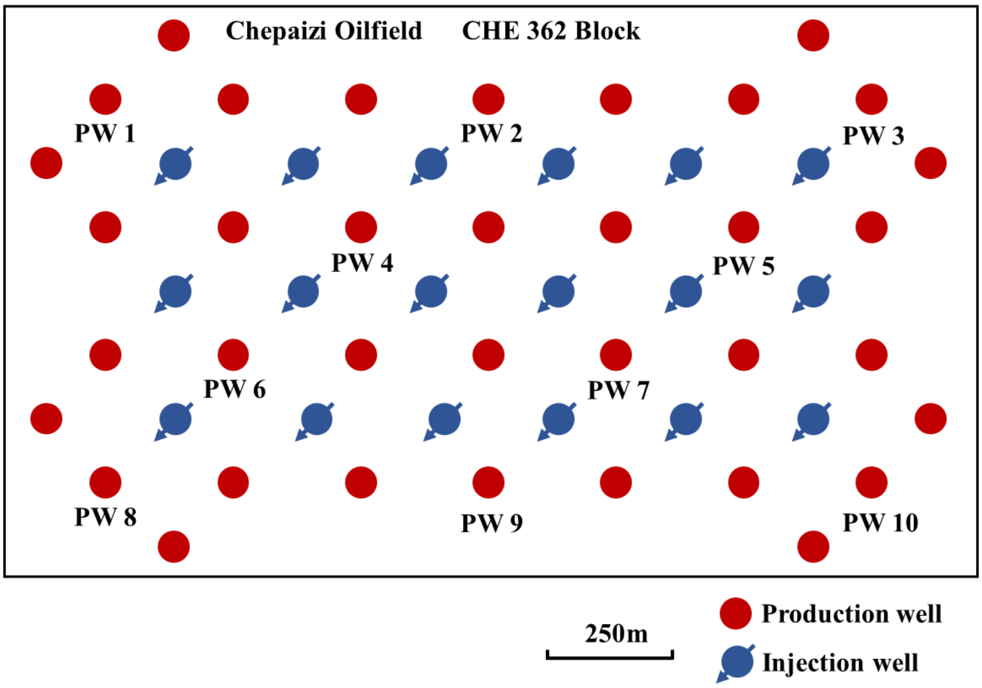

Figure S1. The distribution of the oil wells. The distance between the oil wells is roughly same. Blue represents the water injection well. Red represents the production well. Samples are collected from PW 1 - PW 10 for this study.

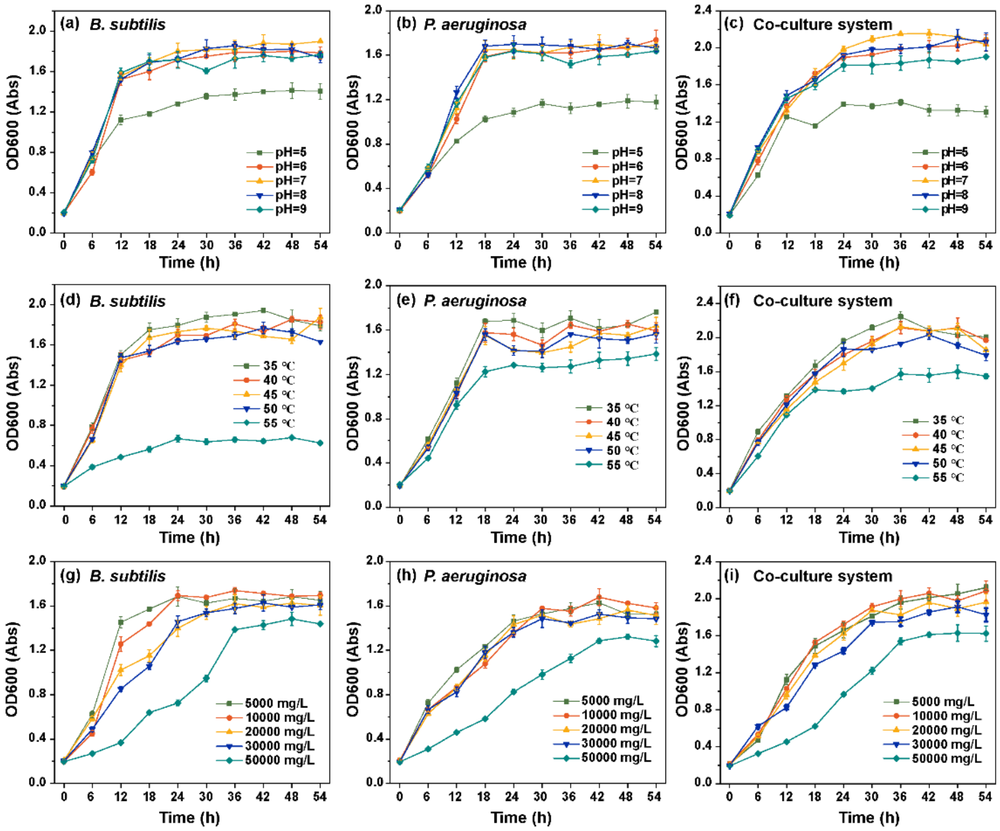

Figure S2. Growth performance of (a) *B. subtilis*, (b) *P. aeruginosa*, (c) co-culture system in sucrose inorganic salt medium of different pH. (d) *B. subtilis* (e) *P. aeruginosa*, (f) co-culture system in sucrose inorganic salt medium at different temperatures. (g) *B. subtilis*, (h) *P. aeruginosa*, (i) co-culture system in sucrose inorganic salt medium with different salinity.

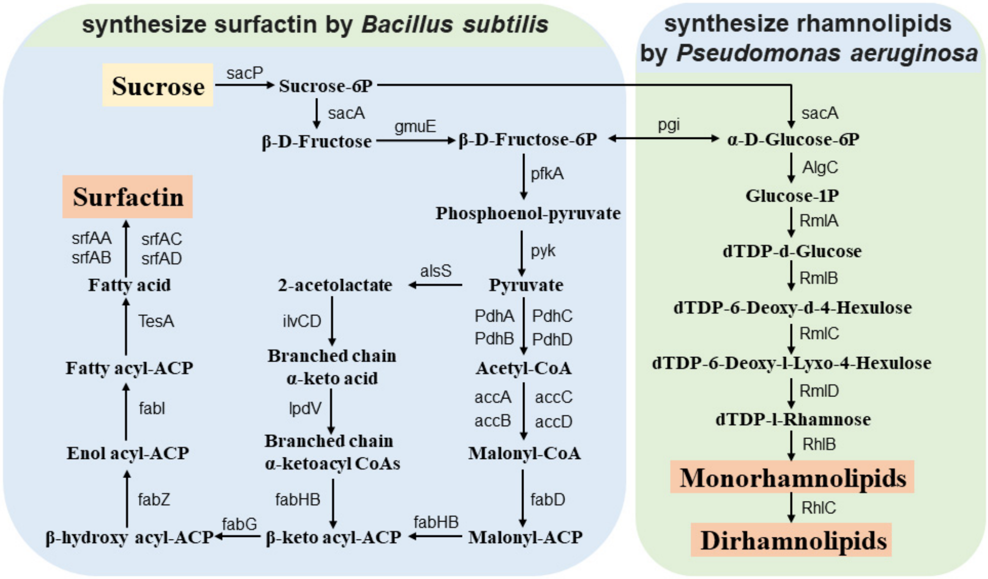

Figure S3. Metabolism of sucrose by *B. subtilis* and *P. aeruginosa* co-culture system to produce biosurfactants.

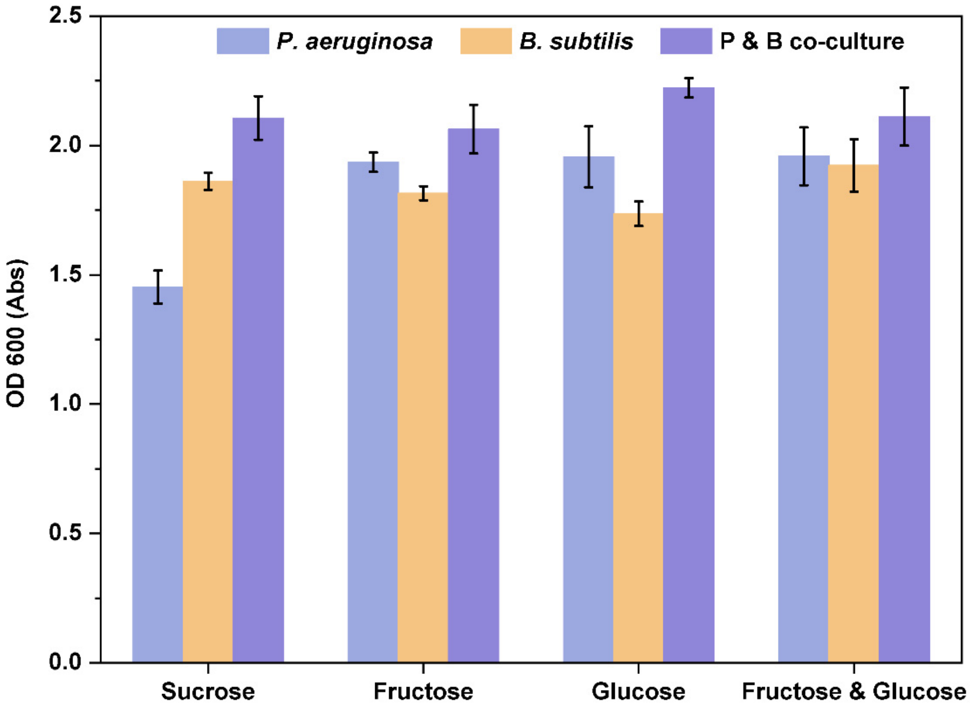

Figure S4. Growth activity of *B. subtilis* and *P. aeruginosa* in separate and co-culture systems in different carbon sources
